# The six most essential questions in psychiatric diagnosis: a pluralogue. Part 4: general conclusion

**DOI:** 10.1186/1747-5341-7-14

**Published:** 2012-12-18

**Authors:** James Phillips, Allen Frances, Michael A Cerullo, John Chardavoyne, Hannah S Decker, Michael B First, Nassir Ghaemi, Gary Greenberg, Andrew C Hinderliter, Warren A Kinghorn, Steven G LoBello, Elliott B Martin, Aaron L Mishara, Joel Paris, Joseph M Pierre, Ronald W Pies, Harold A Pincus, Douglas Porter, Claire Pouncey, Michael A Schwartz, Thomas Szasz, Jerome C Wakefield, G Scott Waterman, Owen Whooley, Peter Zachar

**Affiliations:** 1 Department of Psychiatry, Yale School of Medicine, 300 George St, Suite 901, New Haven, CT, 06511, USA; 2 Department of Psychiatry and Behavioral Sciences, Duke University Medical Center, 508 Fulton St, Durham, NC, 27710, USA; 3 Department of Psychiatry and Behavioral Neuroscience, University of Cincinnati College of Medicine, 260 Stetson Street, Suite 3200, Cincinnati, OH, 45219, USA; 4 Department of History, University of Houston, 524 Agnes Arnold, Houston, 77204, USA; 5 Department of Psychiatry, Columbia University College of Physicians and Surgeons, Division of Clinical Phenomenology, New York State Psychiatric Institute, 1051 Riverside Drive, New York, NY, 10032, USA; 6 Department of Psychiatry, Tufts Medical Center, 800 Washington Street, Boston, MA, 02111, USA; 7 Human Relations Counseling Service, 400 Bayonet Street Suite #202, New London, CT, 06320, USA; 8 Department of Linguistics, University of Illinois, Urbana-Champaign, 4080 Foreign Languages Building, 707 S Mathews Ave, Urbana, IL, 61801, USA; 9 Duke Divinity School, Box 90968, Durham, NC, 27708, USA; 10 Department of Psychology, Auburn University Montgomery, 7061 Senators Drive, Montgomery, AL, 36117, USA; 11 Department of Clinical Psychology, The Chicago School of Professional Psychology, 325 North Wells Street, Chicago, IL, 60654, USA; 12 Department of Psychiatry, Institute of Community and Family Psychiatry, SMBD-Jewish General Hospital, McGill University, 4333 cote Ste. Catherine, Montreal, QC, H3T1E4, Canada; 13 Department of Psychiatry and Biobehavioral Sciences, David Geffen School of Medicine at UCLA, 760 Westwood Plaza, Los Angeles, CA, 90095, USA; 14 VA West Los Angeles Healthcare Center, 11301 Wilshire Blvd, Los Angeles, CA, 90073, USA; 15 Department of Psychiatry, SUNY Upstate Medical University, 750 East Adams St., #343CWB, Syracuse, NY, 13210, USA; 16 Irving Institute for Clinical and Translational Research, Columbia University Medical Center, 630 West 168th Street, New York, NY, 10032, USA; 17 New York Presbyterian Hospital, 1051 Riverside Drive, Unit 09, New York, NY, 10032, USA; 18 Rand Corporation, 1776 Main St Santa Monica, California, 90401, USA; 19 Central City Behavioral Health Center, 2221 Philip Street, New Orleans, LA, 70113, USA; 20 Center for Bioethics, University of Pennsylvania, 3401 Market Street, Suite 320, Philadelphia, PA, 19104, USA; 21 Department of Psychiatry, Texas A & M College of Medicine, 4110 Guadalupe Street, Austin, TX, 78751, USA; 22 Silver School of Social Work, New York University, 1 Washington Square North, New York, NY, 10003, USA; 23 Department of Psychiatry, NYU Langone Medical Center, 550 First Ave, New York, NY, 10016, USA; 24 Department of Psychiatry, University of Vermont College of Medicine, 89 Beaumont Avenue, Given Courtyard N104, Burlington, VT, 05405, USA; 25 Institute for Health, Health Care Policy, and Aging Research, Rutgers, the State University of New Jersey, 112 Paterson St, New Brunswick, NJ, 08901, USA

## Abstract

In the conclusion to this multi-part article I first review the discussions carried out around the six essential questions in psychiatric diagnosis – the position taken by Allen Frances on each question, the commentaries on the respective question along with Frances’ responses to the commentaries, and my own view of the multiple discussions. In this review I emphasize that the core question is the first – what is the nature of psychiatric illness – and that in some manner all further questions follow from the first. Following this review I attempt to move the discussion forward, addressing the first question from the perspectives of natural kind analysis and complexity analysis. This reflection leads toward a view of psychiatric disorders – and future nosologies – as far more complex and uncertain than we have imagined.

## General conclusion

In concluding this multi-part article, let me begin once again with a brief review of what we have already covered. For the full text of the General Introduction to the entire article, the reader is referred to Part 1 [1]. The General Introduction reviewed the history of the article, which originated in a critique by Robert Spitzer and Allen Frances, Chairmen respectively of the DSM-III and DSM-IV Task Forces, over the ongoing work of the DSM-5 Task Force and Work Groups. In a series of articles and blog postings in *Psychiatric Times,* Frances (at times with Spitzer) carried out a sustained critique of the DSM-5 work in which he focused both on issues of transparency and issues of process and content [2-15].

In the course of this debate over DSM-5 I proposed to Allen in early 2010 that we use the pages of the Bulletin of the Association for the Advancement of Philosophy and Psychiatry (of which I am Editor) to expand and bring more voices into the discussion. This led to two issues of the Bulletin in 2010 devoted to conceptual issues in DSM-5 [16,17]. (Vol 17, No 1 of the AAPP Bulletin will be referred to as Bulletin 1, and Vol 17, No 2 will be referred to as Bulletin 2. Both are available at http://alien.dowling.edu/~cperring/aapp/bulletin.htm.) Interest in this topic is reflected in the fact that the second Bulletin issue, with commentaries on Frances’ extended response in the first issue, and his responses to the commentaries, reached over 70,000 words.

Also in 2010, as Frances continued his critique through blog postings in *Psychiatric Times*, John Sadler and I began a series of regular, DSM-5 conceptual issues blogs in the same journal [18-37].

With the success of the Bulletin symposium, we approached the Editor Emeritus of PEHM, Michael Schwartz, and then the Editor, James Giordano, about using the pages of PEHM to continue the DSM-5 discussion under a different format, and with the goal of reaching a broader audience. The new format would be a series of “essential questions” for DSM-5, commentaries by a series of individuals (some of them commentators from the Bulletin issues, others making a first appearance in this article), and responses to the commentaries by Frances. Such is the origin of this article. (The general introduction, individual introductions and conclusions to the separate parts, and general conclusion, are written by this author (JP), the responses by Allen Frances.

For this exercise we have distilled the wide-ranging discussions from the Bulletin issues into six questions: 1) the nature of a mental disorder; 2) the definition of mental disorder; 3) the issue of whether, in the current state of psychiatric science, DSM-5 should assume a cautious, conservative posture or an assertive, transformative posture; 4) the role of pragmatic considerations in the construction of DSM-5; 5) the issue of utility of the DSM – whether DSM-III and IV have been designed more for clinicians or researchers, and how this conflict should be dealt with in the new manual; and 6) the possibility and advisability, given all the problems with DSM-III and IV, of designing a different diagnostic system. Part 1 [1] of this article covered the first two questions, Part 2 [38] the second two questions, and Part 3 [39] the final two questions. This text, Part 4, contains the general conclusion.

We can fairly assume from the preceding discussions that the DSM is at a crossroads. The promise of DSM-III has not been realized, and where we go with DSM-5 and beyond is unclear. This has left us with a mix of voices in the above commentaries that are at times harmonious and at times cacophonous. On this note of concordance and contention we could despair of ever sorting out our nosology, or we might focus instead on the agreements where we find them, take the disagreements as occasions for further discussion, and try to move forward.

Let me first review Allen Frances’ position and his exchanges with his commentators, and then move to the implications of this discussion for DSM-5 and the future.

As all know, Frances did yeoman’s work with DSM-IV and has been in open disagreement with much of the DSM-5 process. As Chief of the DSM-IV Task Force, he is in a unique, privileged, and controversial position to be commenting on DSM-5.

### Question 1

The first question, that of the conceptual status of psychiatric disorder and their diagnoses, is the central question in this article. For that reason, the discussion of this question involves more commentary that the other five questions, and also for that reason, the other five questions in one way or another follow from it. In addressing this question of conceptual status and the five possible responses to it, Frances begins, in the terminology of his umpire metaphor, by informing us that “none of the five umpires is completely right all of the time. And none is totally wrong all of the time. Each has a season and appropriate time at the plate.” He also notes that at the time of DSM-III the biological Umpire 1 and the anti-biological Umpires 3 and 5 were the main players in the debate over the conceptual status of psychiatric disorders, while, in contrast, the nominalist Umpire 2 and the pragmatic Umpire 4 have assumed increased importance in recent years.

In attempting to explain this shift Frances points to psychiatry’s failures to fulfill the promises of DSM-III. We have been unable to validate the DSM categories with the Robins/Guze criteria, and there is a poor match-up of DSM categories with findings in neuroscience and genetics. For Frances this failure of biological psychiatry to realize its expectations moves him toward the second (nominalist) and fourth (pragmatic) umpire positions. With the nominalist, second umpire position, he assumes a middle ground between simple realism and pure social construction, arguing that there are real people suffering from real psychopathology, but that our way of naming and defining the psychopathology may not accurately represent the biological reality of those conditions. Regarding the pragmatiist, fourth umpire position, he insists on a pragmatic, practical dimension in the construction and use of diagnostic categories. We need to be cautious with the terminology in the sense that calling himself nominalist and pragmatic does not mean for Frances that there are no diseases out there (the constructionist, third-umpire position). Although he doesn’t use Peter Zachar’s language of practical kinds [40] or Zachar and Kenneth Kendler’s language of anti-essentialism [41], his position is consistent with theirs. Finally, we need to add Frances’ caution that there is something to be learned from all five positions.

In view of the complexity of the conceptual question, it is not surprising that commentators offer a range of responses, often not confining themselves to one of the five umpire alternatives. Nassir Ghaemi locates himself in the realist Umpire 1 position, while Michael Cerullo places himself in a modified Umpire 1 position, and Jerome Wakefield finds a home in an Umpire 1.5 “humble realism.” With philosophical distinctions Claire Pouncey joins Allen Frances in the nominalist Umpire 2 position. Gary Greenberg is comfortable representing the constructivist Umpire 3 spot. Harold Pincus emphasizes the pragmatic considerations of Umpire 4 and thus joins Frances there. Thomas Szasz has long represented the anti-psychiatry Umpire 5 position and continues to do so here. Peter Zachar and Steven Lobello join Frances in some combination of the Umpire 2 & 4 positions. Joseph Pierre notes that various psychiatric disorders require different positions, and Elliott Martin comments on the interference of outside forces on the entire process.

For my part I have found the nominalist/pragmatic compromise reasonable. It recognizes that there are psychiatric diseases, that our current set of categories represents them very imperfectly, and that in this circumstance we need to pay close attention to the practical use of the categories.

### Question 2

The second question is understandably related to the first. If you can’t decide what a psychiatric disorder is, you’re certainly going to have a hard time formulating a definition of psychiatric disorder. Hannah Decker captures the flavor of this dilemma in her account of the controversy over definition in the pre-DSM-III era. In his struggles with both the anti-psychiatrists and the psychologists Robert Spitzer lost his battle to define psychiatric illness as a medical disorder. Both conceptual and political issues defeated his effort. The struggle over definition continues into the present, with our commentators covering the range of no definition to precise definition.

In this discussion Allen Frances can certainly claim more experience than most through his personal efforts to deal with the issue of definition in DSM-IV. His experience has left him both discouraged at the possibility of getting it right and dispirited at the prospect of putting too much effort into the project. As he muses: “Humpty Dumpty: ‘When I choose a word it means just what I choose it to mean’. When it comes to defining the term ‘mental disorder’ or figuring out which conditions qualify, we enter Humpty's world of shifting, ambiguous, and idiosyncratic word usages. This is a fundamental weakness of the whole field of mental health.”

Nonetheless, Frances is not willing to abandon the notion of including a definition in the diagnostic manual, however flawed that might be. Among our commentators he is closest to Pierre’s compromise solution of a flawed definition, and he would probably support the effort of Stein et al. [42], which might well become the official definition in DSM-5. Our other commentators assume positions at the extremes of the Frances/Pierre compromise. At one extreme Warren Kinghorn sees no need for a definition of mental illness, while at the other extreme Jerome Wakefield offers his clearly formulated, extensively published definition in terms of harmful dysfunction. Finally, John Chardavoyne suggests the notion of a developing, changing definition.

For my part, I continue to feel that in the absence of a consensus regarding the conceptual status of psychiatric disorders and diagnoses, any definition will be imperfect; and that leads me toward a loose, Wittgensteinian approach to definition, as indicated earlier.

### Question 3

As was the case with the second question (that of definition), the third question – whether to assume a conservative or assertive posture in making changes in the new manual – follows from Question 1, that of the conceptual status of psychiatric entities. This issue, however, is different from that of definition. For the issue for definition was: in the face of conceptual uncertainty, should we attempt a definition or not? The issue with the third question is: in the face of the same conceptual uncertainly, should we be modest or aggressive in the construction of the new manual?

Allen Frances has been very assertive in defending a conservative approach to change in DSM-5. He argues rather straightforwardly that, if current science does not match well with the existing DSM categories, and altering the categories will not improve that match, why change them? Why not leave them alone until science, as in the NIMH Research Domain Criteria (RDoC), offers us guidance as to how to change them? And he makes this argument in bold for proposed new diagnoses with minimal scientific foundation such as Attenuated Psychosis Syndrome.

In arguing for a conservative approach Frances calls on his experience in DSM-IV, where seemingly small changes in diagnostic categories led to significant, unexpected consequences. He cites the DSM-IV experience with the inclusion of Bipolar II and Asperger's, and a rewrite of the criteria for ADD. “Each decision was made for excellent reasons that withstand the test of time – but each led to an unexpected frenzy of diagnostic enthusiasm that far overshot the mark beyond the useful purpose of the DSM IV intention.”

In addressing the commentaries on this question of conversatism, we should recall that virtually all discussants – in this article, in the Bulletin discussions, and in the article by Regier and colleagues cited in the general introduction – agree on a number of conclusions about the DSMs: that the DSM-IV categorical profiles often do not adequately reflect the heterogeneity of presentation in individuals grouped under a particular category, that the operationally defined categories, while achieving the reliability that was their goal and in that way facilitating research across different settings, also inhibit research [43] by constricting it to the boundaries of the diagnostic criteria, that the diagnostic constructs create a high rate of comorbidity, as well as a high rate of NOS diagnoses, that most of the diagnoses fail the test of the original Robins and Guze criteria [44], as well as the additional Kendler [45] validator, that current findings in genetics and neuroscience do not match up with the DSM-IV categories, and finally that the research findings of molecular genetics and neuroscience regarding psychopathology are at this point quite unsettled [46].

What is of great interest in Question 3 is that the same set of facts and the same conceptual indecision lead commentators to opposite conclusions. Proceeding from this starting point Michael Cerullo concludes, like Frances, that the current uncertainty of psychiatric science does not warrant substantial change in the new manual, while Scott Waterman argues that that same uncertainty necessitates such change. We gain some insight into this difference by looking at some of the details. Waterman recommends some changes – e.g., eliminating the axial system and paring down the number of categories –that, whatever their merits, do not address the flaws in DSM-IV stemming from its inadequate scientific foundation. When he addresses the latter, he says at one point, that “[t]axonomy cannot lead conceptual innovation but can only aspire to reflect it.” But he then says a little further on: “Those diagnostic entities that remain should both describe and help us investigate the phenotypes, etiopathogeneses, prognoses, and treatment responses of the patients so categorized.” Here then is the controversy. Given that the science is not in place to allow significant redescription of psychiatric categories, should the categories be reformulated in a manner to facilitate further, clarifying research? Frances has responded to this question by saying that “[w]e don't really know which changes would improve science” and “[c]hanges may retard science by making previous findings incompatible with new ones.”

In another section of the article Joseph Pierre entered this discussion by stating (and reflecting the first part of Waterman’s argument): “Diagnostic revision should follow, not precede, etiologic discoveries – in other words, a new DSM-5 needs etiologic discoveries, but etiologic discoveries do not need a new DSM.” In this matter of new etiologic discoveries we should recognize a conflict over diagnostic criteria only partially touched on by the discussants. On the one hand the criteria are needed for reliability among researchers; on the other hand it is now commonly recognized that the same criteria impede research by confining researchers to study groups defined by the criteria. The only proposal we have had for overcoming this conflict has been that of the NIMH RDoC group – a proposal to ignore the DSM categories and criteria entirely in developing their foci of investigation.

Frances is less clear about his conservative position when it involves diagnoses such as the paraphilias that are more value-laden, that are mostly the judgments of public morality, and whose status as psychiatric disorders will not be decided by future science. In defense and explanation of DSM-IV, he offers the historical context in which he and his Task Force felt it was important to maintain the high-threshold standard for change in the case of all diagnoses. He acknowledges that this may have resulted in leaving diagnoses in place that would better have been “sunsetted.”

In his commentary Andrew Hinderliter takes up the other side of this question on the value-based diagnoses, and makes a cogent argument for questioning and removing some of the paraphilias.

For my part I am in agreement with Frances, as well as Cerullo and Pierre, that the current state of psychiatric science warrants a conservative approach to change in DSM-5, and that the limitations in our knowledge cannot support the aggressive approach advocated by Waterman. These arguments become weaker, however, when it comes to the paraphilias, where values predominate and decisions won’t be decided by scienfitic research.

### Question 4

The fourth question, that of pragmatic considerations in developing a psychiatric nosology, once again connects to the questions that precede it, and ultimately to Question 1 – the conceptual status of our nosology. We have already dealt with the shaky conceptual foundations of the diagnostic categories, as well as some of the consequences that follow from that – the problems in developing an adequate definition of psychiatric illness, and the necessary cautions in making unscientifically based changes in the new manual. With the current question we add another consideration into our discussion: whether, in the face of the weak science that undergirds the diagnostic manual, we should pay particular attention to the practical effects of any envisioned changes.

Everyone participating in this discussion has responded unambivalently: Of course, how could we not consider the practical effects of our diagnostic constructions? Allen Frances frames this response in the terminology of the Hippocratic maxim: *Primum non nocere* – First, do no harm. Or in more contemporary language: whatever changes you make in the construction of a new diagnostic manual, don't lose track of the fact that your goal is the treatment of psychiatric patients; consider whether your modifications will cause more benefit than harm.

A question now arises in this discussion: is there a conflict between science and pragmatism? Shouldn’t the major decisions be based on science rather than pragmatic considerations. One could argue, for instance, that the goal of a diagnostic manual is to identify and describe the diseases in the best possible manner, without regard for how the manual might be used by various user groups, or what effect it might have on patients. That is certainly not the stated goal of the DSM-IV and its predecessors, and it would probably be difficult to find someone to argue this position.

Frances and the commentators all agree that pragmatic issues play a role in developing a nosology. Frances points out that the DSM has an enormous effect on how everything works in the mental health world (who gets diagnosed, how they are treated, who pays for it, whether someone can be involuntarily committed, etc.), as well as in public policy (allotment of resources, handling of sex offenders in the legal system, etc.). The four commentators add further thoughts to the science/pragmatics discussion. Warren Kinghorn points out that after you decide to allow for pragmatic considerations, you then have to decide whom you’re going to empower to make the pragmatic decisions (he could have added that we have the same problem in deciding whom we will empower to evaluate the science). Douglas Porter notes that the science/pragmatics distinction is somewhat artificial in that the science itself is so value/laden. Joel Paris adds further that the science is so inadequate that we have no choice but to make decisions on other than a scientific basis. Finally, Joseph Pierre reminds us that in the consulting room we are focused on the suffering person in front of us, and pay little attention to “scientific” factors such as the number of criteria the person meets for a particular diagnosis.

Let me now summarize some of the circumstances in which the dialectic of science and pragmatics is operative in developing a psychiatric nosology.

First, as indicated above, Allen Frances describes in a general way the effects of the nosology on all aspects of both clinical mental health care and public policy regarding mental health issues.

Second, if the scientific basis of a putative category is weak enough to question whether it should be in the manual or not, bear in mind that putting it in the manual will mean more treatment for that group of now officially labeled individuals. For instance, the storm over Attenuated Psychosis Syndrome was in large measure provoked by the fact that outcome studies for this putative diagnosis were showing a 66% rate of false positives for progression to schizophrenia or other serious psychosis.

Third, to the extent that the scientific basis of a diagnostic category is weak, issues of practical concern will play a greater role in the construction of the category. For instance, if the category is shaky, should you emphasize specificity over sensitivity – better to have false negatives than false positives?

Fourth, treatment side effects should probably play a role in sensitivity/specificity balance. For example, if the appropriate treatment is a neuroleptic, with potentially significant side effects, should we again err on the side of specificity over sensitivity – fewer false positives in the face of unnecessary use of neuroleptics.

Fifth, as noted above, diagnoses that are highly value-laden, e.g. the paraphilias, don’t readily lend themselves to a scientific determination as to their disease status. Decisions about their inclusion in a nosology will inevitably involve considerations other than scientific ones.

Sixth, and finally, *all* diagnostic categories, however solid their scientific foundation, will involve the issue of sensitivity and specificity in their diagnostic criteria. The example I used earlier was that of major depression. Increase the number of required criteria and you increase specificity and produce false negatives. Decrease the number of required criteria and you increase sensitivity and produce false positives. The use of dimensional measures appears to ameliorate this problem by allowing for every degree of the condition, but in fact it merely moves the pragmatics of sensitivity/specificity to another question: at what point on the scale do we say, no major depression.

With this question, then, Frances, the commentators, and this author are all in agreement regarding the appropriate inclusion of pragmatic issues in the construction of psychiatric diagnoses. The commentators, rather than presenting disagreements, focus more on the fine points of the discussion – e.g. Kinghorn pointing to the question as to who is empowered to make the pragmatic decisions, Porter adding the same question as to who is empowered to evaluate the value-laden science, Paris emphasizing the limitations of our science, and Pierre emphasizing that the fact of dealing with the person in one’s consulting room nudges the discussion toward a pragmatic focus on that individual as opposed to a focus on trying to get the DMS-IV diagnosis right.

### Question 5

Discussion of the fifth question, utility, has had two foci: the general structure of DSM-IV, and the primary, proposed innovation of DSM-5, dimensional measures. The first focus is heralded by the statement from the Introduction to DSM-IV:

“The utility and credibility of *DSM-IV* require that it focus on its clinical, research, and educational purposes and be supported by an extensive empirical foundation. Our highest priority has been to provide a helpful guide to clinical practice. We hoped to make *DSM-IV* practical and useful for clinicians by striving for brevity of criteria sets, clarity of language, and explicit statements of the constructs embodied in the diagnostic criteria. An additional goal was to facilitate research and improve communication among clinicians and researchers. [47], p. xv].”

Regarding this focus on utility in DSM-IV, I pointed out in the earlier introduction to Question 5 that the question, “Is there a conflict over utility in the DSMs?,” contains in fact three questions: is there a conflict among the manual’s various goals?; what goal or purpose is served best by the manual?; and would we be better off with more than one manual? Given that Allen Frances was the primary architect of DSM-IV and thus responsible for the above-quoted statement from its Introduction, it is not surprising that, while acknowledging possible tensions among the different goals of the manual, he comes to the defense of a single manual. He writes:

“Psychiatric classifications are like maps – and like maps there is no single best way of charting the territory. Depending on our purpose, we may prefer to use a geological map, or a political map, or a topographical map, or an economic map, or a climate map-or some combination. Similarly, it might have made sense to have different and complementary DSMs – one for clinicians, another for researchers, and others for education, for forensics, for billing, for gathering statistics, and so on. The DSM we have is a common denominator, with both the strengths and the weaknesses that derive from attempting to serve so many different masters…”

“DSM-IV does none of its jobs perfectly and its awkward fit certainly creates a variety of problems. Some clinicians refuse to learn DSM and stick to their own personal prototypes of disorders. Many epidemiological researchers ignore the requirement for clinical significance before making a psychiatric diagnosis and therefore report ridiculously high rates of mental illness in the general population. Some students take the DSM descriptions too literally and lose the patient as they evaluate the criteria. Lawyers often find loopholes because the language of DSM is frustratingly below legal requirements for precision. And so on.”

“But the unifying and synthesizing whole of DSM-IV is still worth much more than would be the accumulated sum of its individual parts. However imperfect, the DSM's special value is as a common denominator that avoids a Babel and is good enough (if admittedly not best) at each of its jobs.”

In these comments Frances deals with the three-part question posed above by granting a potential conflict among DSM-IV’s stated goals (part 1), making a strong argument for maintaining a single manual (part 3), and scanting the question as to what goal is best served by the manual. Assuming that he would still endorse the statement from the Introduction to DSM-IV, we could assume he would still place clinical practice as the primary goal of the manual.

Unlike the discussion in the other questions, where some or most of the commentators were in agreement with Frances’ argument, in the question of utility we have all three commentators lined up against him: all recognizing the clinical/research conflict, all agreeing that DSM-IV is prejudiced toward research, and two of the three leaning toward separating clinical and research diagnostic documents. While in agreement that there is a conflict between the clinical and research goals of the DSM, the three commentators offer different perspectives on the conflict.

Owen Whooley adopts Aristotle’s distinction between theoretical and practical knowledge (*episteme* and *phronesis*) and argues for a deep, metaphysical divide between the research and clinical goals of DSM-III/IV – in Aristotelian terms a divide between the search for universal laws versus individualized care of the particular patient.

Joseph Pierre focuses on the DSM architects’ own confusion regarding the goal and use of the manual. Although the diagnostic criteria are claimed to have a primary clinic use, their real goal is in promoting research. But in fact, as pointed out above, they impede research as much as they facilitate it. Pierre points out that the most promising research, that of the RDoC, is being done outside the confines of the DSM.

Finally, Aaron Mishara and Michael Schwartz point to the clinical/research conflict as a consequence of basing DSM-III on a Hempelian scientific model; they argue that a DSM designed with the ideal-type structure they advocate would eliminate the clinician/researcher split and would in fact serve the two groups equally well.

The second focus of the utility question is the introduction of dimensional measures into DSM-5. Regier and colleagues announced this innovation in the following manner:

“The single most important precondition for moving forward to improve the clinical and scientific utility of DSM-5 will be the incorporation of simple dimensional measures for assessing syndromes within broad diagnostic categories and supraordinate dimensions that cross current diagnostic boundaries. Thus, we have decided that one, if not the major, difference between DSM-IV and DSM-5 will be the more prominent use of dimensional measures in DSM-5 [48] (see also [49]).”

These authors assure us once again that these dimensions will provide clinical as well as scientific utility. The proposed dimensional measures raise two questions: should they even be in the manual, and who will use them. The questions raised above about who will use them are apposite here. Given their undemonstrated scientific status, Frances challenged their introduction into DSM-5 in Bulletin 2, Whooley challenges it in this section (drawing agreement from Frances), and Paris challenges it in the context of Question 6. First and colleagues have argued that any change in the existing manual should use clinical utility as a criterion of change [50], and First has expressed concern over the dubious clinical utility of the proposed measures [51].

To include my opinion in this discussion, I can certainly sympathize with Frances’ argument for a single manual, but I also agree with the arguments of the commentators regarding the conflict between clinical and research utility (and have written about the conflict [25]). Whooley and Pierre might well argue for separate manuals, although they don’t quite take their arguments to that conclusion. I would opt for a variation on the simplification proposed by Mishara and Schwartz: to wit, the current manual with its prototypal/ideal-type categories, and the diagnostic criteria moved to the back of the manual. In that manner the manual would be clearly designed for clinicians, and the appendix with diagnostic criteria would be available for researchers. Thus one manual, but a manual that was genuinely useful for clinicians and that placed specific research needs in an appendix.

We might make the same point with respect to the dimensional measures. They have no defenders in this discussion, and thus no dispute between Frances and his commentators. They could be confined to the same fate as the diagnostic criteria – one more appendix at the back of the manual. They would be there for whomever might want to use them, but they wouldn’t be in the face of clinicians who would just ignore them anyway.

### Question 6

The second question asked whether the multiple problems of DSM-IV warrant a conservative or aggressive attitude toward change in DSM-5. The sixth and final question takes this issue to its ultimate interrogation: should we consider a major overhaul or even another manual? Consistent with his response to Question 2, Frances argues that the state of psychiatric science dictates minimal change, not the “paradigm shift” proposed by the DSM-5 architects, and not any other form of major overhaul. Around the question of alternate systems he writes:

“Every month or so, someone (usually very smart and passionate) sends me a detailed proposal for a new diagnostic system offered as an alternative to the jumbled, pedestrian, atheoretical, and purely descriptive method used in DSM. The new system is invariably theory driven, clever, neat, and plausible. Surely, it is quite easy to be more coherent than a DSM that consists of a jumble of disorders gathered together largely through a historical accreting process based mostly on clinical observation and descriptive research – without an underlying theory or deep knowledge of causality.”

Frances argues that none of the alternate systems on offer is anywhere near ready for actual use. He also invokes the NIMH Research Domain Criteria project that promises to change the scientific landscape of psychiatry in the future. Pending findings from that endeavor, which may indeed warrant a significant refashioning of the DSM, we should hold tight and await the return of the RDoC jury.

We can sort the possible responses to this question into three groups: alternative systems already in development, alternative nosological systems developed by the commentators themselves, and major restructuring proposals for the DSM. For a representative of the first group we have First’s presentation of the NIMH Research Domain Criteria Project (RDoC). In the second group belongs Pies’ proposal (along with those of Hayes, Mender, Mishara and Schwartz, Peled, and Pies in Bulletin 2). And to represent the third group we have Paris’ discussion of the thrust toward dimensional measures in DSM-5 (also covered by Whooley in the preceding question).

The first commentary follows on Frances’ proposal to await results from the NIMH RDoC project. Michael First provides a clear description of the project, emphasizing that this is research project, not an alternative diagnostic manual. But it is a research project whose findings may significantly affect all DSMs that follow in its wake. In a second commentary Ronald Pies reviews his effort at imagining an alternative diagnostic system, described more thoroughly in Bulletin 2. His proposal involves two innovations: basing the system on prototypal diagnostic constructs, and dramatically reducing the number of diagnoses from several hundred to a large handful. Although structured differently, his proposal is quite consonant with that of Mishara and Schwartz in the emphasis on prototypes and a limited number of categories. Finally, Joel Paris tackles the major innovation of DSM-5, the introduction of a variety of dimensional measures. His critique overlaps with some of the discussion in the previous question on utility - for the obvious reason that the introduction of such measures involves both questions, utility and alternate systems. He is in agreement with previous discussion of this topic – indeed, we have not had a positive response, either from Frances or any of the commentators, toward the proposed dimensional measures. Paris also includes a suggestion, again harkening back to the discussion of Question 5, that the manual might work better by being split into two: a shorter version for clinicians and a more detailed version for researchers.

With question 6 I can in some fashion agree with everyone. Allen Frances makes a convincing case that, pending more definitive science than currently available, we should stick with the DSM that we have, but, in agreement with Joel Paris, without the dimensional measures currently planned. Frances, like other commentators, also makes a case for awaiting findings of the NIMH Research Domain Criteria project as described by Michael First. Of course none of us knows whether the RDoC project will produce the ‘paradigm shift’ it promises for psychiatric nosology. Contra Frances, I also agree with the arguments that the current manual is tilted toward research, and that it is too cluttered with diagnostic categories. I agree with the proposals of Mishara and Schwartz, and of Pies, to reduce the number and focus on prototypes. My own addition would be to move the diagnostic criteria (along with the dimensions) to the back of the manual. Frances defends the retention of operational definitions and diagnostic criteria. The argument for retention remains the original argument for reliability. The argument for dismissal (perhaps to an appendix) is that clinicians don’t use them, they impede as well as enhance research, and they produce reliability without validity, with the inevitable consequence of excessive comorbidity and NOS diagnoses.

### Further considerations: DSM-5 and the future

I indicated at the beginning of this general conclusion that the central question in this article is the first one, the nature of mental disorders, and that the remaining questions are to one degree or another secondary to the first. In these concluding remarks I return to the first question and try to draw out the analysis a bit further. I do this in terms of natural kind analysis and complexity theory.

### Natural kind analysis

First, recall that questions regarding the conceptual status, or nature, of psychiatric disorders stem from failure of DSM-III/IV categories to achieve scientific validity as genuine, scientifically based disease entities. When the categories of DSM-III were defined with diagnostic criteria in 1980, it was assumed that reliability would be first accomplished and that, with further science based on the operationally defined categories, validity would follow. Robins and Guze, representing the Washington U group, had set five criteria or standards of validity (called “phases” of validity in the seminal article) [44], and it was assumed that those standards would be met in the ensuing years. The great shock of the past forty years is that the DSM-III/IV categories have not met the Robins/Guze standards. As Regier and colleagues wrote in 2009, already quoted above,

“The expectation of Robins and Guze was that each clinical syndrome described in the Feighner criteria, RDC, and DSM-III would ultimately be validated by its separation from other disorders, common clinical course, genetic aggregation in families, and further differentiation by future laboratory tests—which would now include anatomical and functional imaging, molecular genetics, pathophysiological variations, and neuropsychological testing. To the original validators Kendler added differential response to treatment, which could include both pharmacological and psychotherapeutic interventions. [48] 645.”

It is worthwhile to do a brief review of the criteria along with the current status of clinical experience and psychiatric research.

1. Clinical description

This includes symptoms aggregating into syndromes, along with other information involved in the common description. Clinical work with the DSM categories has shown a great heterogeneity of presentation, thus defeating the criterion that a valid category should present a uniformly described syndrome.

This phenomenon of heterogeneous presentation should not be a surprise, as it is common in the rest of medicine, where the same disease may present in different ways, and a similar presentation may represent different diseases. For instance, syphilis and streptococcus may each present in a variety of ways (same disease, different presentations), and shortness of breath may represent CHF, pneumonia, or COPD (same presentation, different diseases). The reason this is not a problem for general medicine is that the labeling of something as a disease is not dependent on the clinical presentation, as in psychiatry. We can conclude that, with respect to this standard of validity, psychiatry is in the place of the rest of medicine over 100 years ago. Further, we can note that “clinical description” was a poor choice by Robins and Guze and that, based on the rest of medicine, it was bound to fail.

2. Laboratory studies

The most striking example of the failure of laboratory studies is that we still do not have a clear biological marker for any of our DSM categories [52]. And we have to add that current work in neuroscience, neuroimaging, and genetics has not led to clear patters that match up with the DSM categories [46]. Probably the most alarming findings are in genetic studies, where the mismatch between genetic patterns and DSM categories has become quite clear [53,54]. What now seems almost obvious is that the science is not falling into place because the DSM categories do not represent distinct diseases – or, in the language of genetics, do not represent real phenotypes.

3. Delimitation from other disorders

With this criterion Robins and Guze recognized that the first two criteria might allow for two disorders to overlap with similar description and laboratory findings, and that we would need a way to distinguish them. In strong contrast to meeting this standard, DSM-IV has been plagued with high comorbidity, along with fuzzy boundaries between categories and the use of NOS diagnoses.

4. Follow-up study

The authors acknowledge that this is not a strong criterion, inasmuch as the same condition could have variable outcomes. That has of course been found to be the case with major disorders such as schizophrenia and bipolar disorder.

5. Family study

The authors argued for this as a strong criterion, and indeed, identical twin studies have shown family aggregation for many conditions. The problem is that family studies have shown both a higher familial incidence of the condition of the target patient, but also a high incidence of other psychiatric disorders [55]. And genetic studies have shown similar genetic patterns in a variety of conditions [46,53,56]. Thus, research has in some ways (identical twin studies) supported the DSM categories but in other ways (family and genetic dispersal) has not.

6. *Differential response to treatment

In 1990 Kenneth Kendler proposed a sixth criterion for validity [45]. Unfortunately, clinical experience over the past twenty years has moved rather dramatically against this criterion. Rather than classes of psychopharmacologic agents matching up with particular diagnoses, we have moved into an era of pharmacologic promiscuity in which many agents are being found to be effective for a variety of disorders (e.g., neuroleptics effective as mood stabilizers, SSRIs effective for a great variety of conditions).

If the DSM diagnostic categories have failed to meet the Robins/Guze standards of validity, we are forced to ask, what are these diagnostic categories? Are they anything real? This of course was the question posed as Question 1. Allen Frances responded, with agreement from a significant number of the commentators, that the categories are constructs that do not reflect real disease entities. He quickly added that that is not an anti-realist, anti-psychiatry, purely social constructionist position. Mental illness exists in the real world, and there are a lot of people suffering from it. It’s just that our current diagnostic categories may not be accurate presentations of the mental illness out there. Our diagnostic constructs are simply our best, current effort to describe psychiatric illnesses. These constructs have been shown to be quite inaccurate, and we can expect that with time and more science the diagnoses will more accurately reflect and describe the range of psychiatric illness.

One way to get a further perspective on this question of the DSM categories and the nature of psychiatric illness is through what is called natural kind analysis. The latter refers to the effort to classify the things of the world in a realistic manner that does not depend on human judgment. A cow is a natural kind, a unicorn is not. You may come across a specimen in the real world and declare it to belong to the natural kind, cow. You won’t find anything fitting the natural kind, unicorn. Writing about natural kinds, Ian Hacking remarks: “The canonical examples have been: water, sulphur, horse, tiger, lemon, multiple sclerosis, heat and the color yellow. What an indifferent bunch!” [57], p. 167]. Hacking includes everything that can be classified, but in a more strict sense a natural kind is considered to have an essential structure that can be described by necessary and sufficient conditions. For instance, if something has an atomic weight of 79, that something is gold; if another thing has a molecular structure of H_2_0, that thing is water. In both cases the thing in the world meets necessary and sufficient conditions for being gold or water. John Locke provided an early description of real natural kinds with his distinction between real and nominal essences. The latter category represents our casual manner of classifying things, as in the Hacking quote above. A real essence would be defined in terms of its microstructure. In making this distinction Locke anticipated our modern tendency to define strict natural kind status in terms of the reductionist language of physics. The examples of gold and water are characteristic of this tendency.

In natural kind analysis there is a fundamental divide between strict natural-kind theorists as just described who assign natural-kind status only to entities that can be defined by essential properties and necessary and sufficient conditions, and other theorists who argue that there is a scale of natural kindedness, that something may be a natural kind in a strong or weak sense. In *The Disorder of Things*[58] John Dupré makes a strong case for the second position. “This thesis is an assertion of the extreme diversity of the contents of the world. There are countless kinds of things, I maintain, subject each to its own characteristic behavior and interactions” (p. 1).

In a strict sense all medical and psychiatric conditions would be judged as not natural kinds because in every case designating something as a disease involves a human value judgment. A broken bone may be an objective, strong natural kind, but declaring the broken bone an ailment involves a value judgment that does not inhere in the bone.

It is much more useful to sort out the world – and the world of illness – with degrees of natural kindedness. For instance, HIV infection can be defined with the necessary and sufficient condition of a positive test; Huntington’s disease can be defined with the necessary and sufficient condition of the Huntington gene HTT. These are relatively strong natural kinds. On the other hand, migraine is defined by clinical evaluation; schizophrenia and major depression are defined by symptoms and diagnostic criteria. These latter three are all quite weak natural kinds compared to HIV or Huntington’s disease, and even more so compared to gold or water. There are no necessary and sufficient conditions for calling something migraine, schizophrenia, or major depression. Indeed, if schizophrenia, for instance, turns out be not one disease but rather a cluster of diseases, it may be a natural kind only in a very weak sense.

In dealing with psychiatric disorders, and with the DSM categories, Peter Zachar makes a useful distinction between classical categories and prototype categories [59]. The former refer to essentialist, strict-sense natural kinds. Either something meets the conditions of belonging to the category, or it doesn’t. Prototype categories describe an idealized exemplar of the category and then judge individuals as more or less like the prototype. Individuals don’t meet necessary and sufficient conditions; rather, they are more or less like the ideal exemplar. (Of course we could also say that they meet the necessary and sufficient conditions more or less.) Zachar describes the DSM categories as prototype categories and not natural kinds, since they are based on prototype descriptions of the respective disorders. Technically he is only half right, since in fact the DSM categories are hybrids. They are prototypes in that they all have both prototype descriptions and lists of diagnostic criteria that the individual may meet in varying degrees. But the fact that the criteria all have a minimally sufficient number of criteria for making the diagnosis flips them into a status of classical categories, with necessary and sufficient conditions. Meet the minimal required number of criteria and you have the disorder; don’t meet it and you don’t have the condition.

If we accept a view of natural kinds as existing on a scale of strength and weakness, it follows that phenomena at the weak end of the scale, where membership is not defined by necessary and sufficient conditions, may be grouped in different ways to satisfy different strategies. If this sounds arbitrary, that’s because it is. At this point in time all psychiatric disorders are weak natural kinds, albeit some weaker than others. As weak natural kinds, we will group them as it seems useful for whatever purpose we have in mind. Our groupings will start with the commonalities – the natural kind status – that we do find, and our decisions as to how to proceed from there will depend on what we find most useful for what we are trying to accomplish. It is obvious that some groupings will be useful than others.

We can readily connect this analysis with the discussion in Question 1. Those arguing for a strict realism (Umpire 1) – diagnostic categories accurately describing real psychiatric disorders – can be called the natural kind essentialists in this discussion. Those arguing with Allen Frances that the categories are constructs that point, however poorly, to real psychiatric illness (Umpire 2), are the weak natural kind theorists. Those arguing that the categories are pure social constructions, or that there is no mental illness (Umpires 3 & 5), assume a position that the diagnostic categories have no natural kind status of any kind. Finally, those who argue that the categories serve pragmatic goals (Umpire 3) are again weak natural kind theorists who link up with the second umpire. (Allen Frances, we may recall, declared himself a combination of Umpires 2 & 4 - in that way defining himself as a weak natural-kind theorist on two counts.)

For an example of psychiatric disorders as weak natural kinds, let’s imagine that the current category, schizophrenia, proves, as seems likely, to include a variety of conditions with different genetic patterns, different endophenotypes, different outcomes, and so forth. For a variety of reasons we may decide to retain the supercategory, schizophrenia, or to break it up into five, ten, or fifty separate conditions. There will probably be no one, right answer to the question, how to group or regroup all the individuals that now fall under that category. Which of course is to say that we don’t expect any of the possible outcomes to enjoy the status of strong natural kind with necessary and sufficient conditions like those of Huntington’s disease. A classification based on endophenotypes, such as the RDoC enterprise (to be discussed below), might claim stronger scientific, natural kind status, and for that reason might make a claim for priority in the reclassification of schizophrenia. But that classification might be very unwieldy as well as less desirable for other reasons. At this point we have no idea how we will want to classify today’s schizophrenics in ten years.

Or let’s imagine we decide to remove the paraphilias from the diagnostic manual for a variety of pragmatic and political/societal reasons. They will still remain weak natural-kind conditions or personality types, but for reasons other than scientific ones will no longer be members of the diagnostic manual. In that case it’s hard to imagine any scientific finding that will move the paraphilias into a strong natural kind status requiring us to declare them psychiatric diseases that *must* remain in the manual.

To conclude this discussion of natural kinds, we need to address the NIMH RDoC project. Michael First has provided an excellent description of the project in his commentary on Question 6. The most striking thing about the project is that the research is being conducted outside the confines of the DSM categories. The developers of the project recognize that the effort to validate the DSM categories as real phenotypes has failed. Their work is based on the assumptions that 1) psychiatric disorders originate in disruptions or malfunctions of neural circuitry, and 2) that specific circuitry malfunctions can be linked to specific cognitive and behavioral abnormalities. Their presumption is further that the genetic basis of these malfunctions may be more clearly defined than the complex patterns associated with DSM categories. The target dysfunctions of the RDoC research would then represent genuine endophenotypes. Of course it remains to be seen how successful the RDoC project will be, and whether it will translate into a more scientific nosology than the current DSM. The authors of DSM-5 hold out the promise that the RDoC research will be integrated into DSM-5 and will effectively save it [60].

### Complexity theory

The above analysis examined the nature of psychiatric disorders and diagnoses in terms of natural kind analysis. The conclusion of that examination was that psychiatric disorders are weak natural kinds. We now approach psychiatric conditions from a different perspective, complexity theory. Complexity theory, and the related chaos theory, have been described in a variety of way. For this discussion I will follow the relatively simple and straightforward approach to complexity developed by Bechtel and Richardson [61].

The approach described by Bechtel and Richardson to analyze complex behavior is one of decomposition and localization, or decomposition and reassembly. The system is decomposed into its parts and then understood as a whole determined by the organization of the component parts. They describe three levels of such mechanistic explanation. The first, termed aggregative, describes a system in which each part has its task, and functioning of the whole can be understood in terms of the components working together, each contributing its specific function. The working of ordinary machines like clocks and automobiles can be understood in this bottom-up, aggregative analysis.

Biological systems virtually never permit of such reductive analysis. Bechtel and Richardson describe further levels of complexity to accommodate biologic systems. “Some machines, however, are much more complex: one component may affect and be affected by several others, with a cascading effect; or there may be significant feedback from “later” to “earlier” stages. In the latter case, what is functionally dependent becomes unclear. *Interaction* among components becomes critical. Mechanisms of this latter kind are *complex systems*…In such cases, attempting to understand the operation of the entire machine by following the activities in each component in a brute force manner is liable to be futile” (p. 18).

Analysis of complex systems proceeds through an interplay of analysis and synthesis, bottom-up and top-down approaches. The whole is understood in terms of the parts, but the parts are also understood in terms of the whole. In the simpler form of complex system termed *component system*, the component parts can still be studied independently, despite the fact that their actual functioning will depend on the total organization of the system. In the more complex *integrated system* the component parts lose their independence and can only be studied as components of the integrated system. What they are and how they work will change with the changing organization of the whole system.

Virtually all biologic systems are complex, as are virtually all diseases. Although a disease like Huntington’s disease might be described as a component system, with straightforward Mendelian causality, most psychiatric disorders, to the extent that it is appropriate to describe them as unitary disorders (as opposed to classifications that comprise many separate disorders), are integrated systems. “In *integrated systems*, systemic organization is significantly involved in determining constituent functions” (p. 20).

The problem in recognizing psychiatric disorders as complex, integrated systems is that it is extremely difficult to study them in this manner. Any factor playing a role in the production of the disorder is affected by all the other factors at play. At the extreme we will say that no two individuals will have exactly the same disorder. To illustrate this problematic, I invoke the work of Kenneth Kendler, psychiatry’s premier researcher on the causation of psychiatric illnesses. Kendler has presented a mixed picture in the matter of complexity, arguing strongly for a complexity, decomposition-reassembly, approach to psychiatric etiology [62], and conducting his research in a manner that combines aggregative and integrated styles of analysis. In two articles on developmental models for major depression in women and men, he developed the models through the analysis of multiple risk factors, seen both as direct causal agents and as interacting, mutually influencing causal factors [63,64]. In his recent “The Dappled Nature of Causes of Psychiatric Illness: Replacing the Organic-functional/hardward-software Dichotomy with Empirically Based Pluralism,” [65] he acknowledges his aggregative approach (“Furthermore, for pragmatic reasons, I initially assume an independence of difference-makers that does not exist in nature” [p. 379]) and then analyzes the respective variance of multiple causal factors for schizophrenia, major depression, and alcohol dependence in a rather thoroughly additive manner. He ends the article with a further acknowledgment that the methodology of the research does not do justice to the complexity of psychiatric disorders.

“The results of the empirically based pluralistic analysis of the causes of SZ, MD and AD reinforce the conclusions from a prior essay that the commonly expressed wish to develop an etiologically based nosology for psychiatric disorders is deeply problematic. Psychiatric disorders are a result of multiple etiological processes impacting on many different levels and often further intertwined by mediational and moderational interactions between levels. It is not possible a priori to identify one privileged level that can unambiguously be used as the basis for developing a nosologic system.”

“My call for an empirically based pluralism does not reflect pessimism about the future of research in the etiology of psychiatric disorders. Surely, they are stunningly complex. But having overly simplified views of them, often ideologically driven, has only hampered our field. Following methods of decomposition and reassembly, progress has been made in the scientific understanding of very complex systems. Having a realistic view of the causal landscapes of psychiatric disorders can only help (p. 385)”.

We can take two lessons from Kendler’s experience. The first is that research into psychiatric disorders as complex systems will be very difficult. The second is that the research will involve decisions as to which factors, levels of analysis, etc. to prioritize. The prioritizing of one over the other will result in a different picture of the respective disorder. And of course now we are back with the issues of natural kind analysis – different manners of sorting the field of psychopathology, each potentially equally legitimate, and choices made to serve particular ends.

## Competing interests

MF is an external consultant to the NIMH Research Domain Criteria (RDoC) Project. NG has research grants from Pfizer and Sunovion, and is a research consultant for Sunovion. MS is a consultant for AstraZeneca, Merck, Novartis and Sunovion. Other authors report no competing interests.

## Authors’ contributions

JP(Phillips) wrote the general General Introduction and Conclusion, as well as the introductions to the individual conclusions. AF wrote the Responses to Commentaries. MC, JC, HD, MF, NG, GG, AH, WK, SL, EM, AM, JP(Paris), RP, HP, DP, CP, MS, TS, JW, SW, OW, PZ wrote the commentaries. All authors read and approved the final manuscript.
